# Social media and management of COVID-19 in a developing country: the case of Cameroon

**DOI:** 10.11604/pamj.2021.38.344.25033

**Published:** 2021-04-09

**Authors:** Valirie Ndip Agbor, Larissa Pone Simo, Terrence Beteck Epie

**Affiliations:** 1 Clinical Trial Service Unit and Epidemiological Studies Unit, Nuffield Department of Population Health, University of Oxford, Oxford, United Kingdom,; 2 Department of Clinical Research, Health Education and Research Organization (HERO), Buea, Cameroon,; 3 Clinical Research Education, Networking and Consultancy (CRENC), Douala, Cameroon,; 4 General Practice, Dzeng Sub-divisional Hospital, Centre Region, Cameroon,; 5 Centre for Tropical Medicine and Global Health, Nuffield Department of Medicine, University of Oxford, Oxford, United Kingdom

**Keywords:** COVID-19, social media, Cameroon

## Abstract

Since the first reported case of the coronavirus disease 2019 (COVID-19) in Wuhan China, the virus has spread to every continent, including sub-Saharan Africa. There exist no cure or vaccine for COVID-19. Classic public health approaches such as hygiene and sanitation, and social distancing are the recommended measures to contain the spread of the causative virus. While it is possible to combine strict lockdown measures in some western countries, this is not practical in almost every country in sub-Saharan Africa. In Cameroon, those without symptoms are encouraged to respect measures of hygiene and sanitation, physical distancing, and to wear a mask in public places. Those who develop symptoms are isolated in accredited COVID-19 management centres until they recover. However, the latter strategy is ineffective in containing the local spread of the virus because testing is not robust. Intuitively, the control of the virus in Cameroon depends largely on how engaged the public is in fighting against the virus. Social media can complement the use of community health workers for community or public engagement. In this viewpoint, we discuss how to optimize public engagement, to combat misinformation and to develop a culture for preparedness amidst the COVID-19 pandemic when time and resources are of the essence.

## Commentary

### Introduction

The world has experienced a series of pandemics with the 1918 influenza pandemic being the most severe in recent history. Globally, about 500 million people were infected with the H1N1 virus, which resulted in 50 million deaths [1]. This happened about a century ago in the absence of the communicative technology today´s society relies upon. Information transmission was long and tedious as postal workers, teachers and boy scouts were required to provide educational materials and teach health precautions to the public. Likewise, physicians reported their number of cases daily to the city health department which in turn reported their statistics to the newspapers [2]. This chain of information gave rise to misinformation and falsification.

Today, the world is experiencing the Coronavirus disease 2019 (COVID-19) pandemic which is an infectious respiratory disease caused by the severe acute respiratory syndrome coronavirus 2 (SARS-CoV-2). As of 1 June 2020, about six million cases had been confirmed with over 300,000 deaths [3]. The African region has recorded approximately 100,000 cases and 3,000 deaths since the first case of COVID-19 was reported on the continent [3]. The first case of COVID-19 in Cameroon was recorded on 6 March 2020. By 1 June 2020, a total of 6752 cases of COVID-19 had been reported with 3629 recoveries [4]. A total of 199 deaths were reported, giving a hospital case fatality ratio of 2.9% [4]. Cameroon was the seventh country in Africa with the highest number of reported COVID-19 cases [3]. So far, the virus has spread to all ten regions in Cameroon [4].

Globally, over 2.9 billion individuals use social media [5]. A survey of 25,000 participants showed a 70% increase in internet usage and 61% higher social media engagement over the usual usage rate since the start of the pandemic [6]. WhatsApp and Facebook were the most used messaging social media platforms [6, 7]. The recent spike in social media and internet usage is most likely due to the need to connect with family, friends, and colleagues, and to access reliable information on COVID-19 such as transmission and measures of prevention. Cameroon, like many countries in the African region, uses social media platforms to communicate information to control the pandemic. In Cameroon, there is an estimated 7.8 million internet users with 3.7 million social media users [8].

Social media provides an invaluable opportunity to enhance rapid dissemination of health information necessary to curb the COVID-19 pandemic, and to socially connect people while intensifying physical distancing measures. For example, the United Nations Child Fund is using various social media platforms such as Twitter, Facebook, YouTube, and Instagram to disseminate information on COVID-19 and collect feedback from the public [9]. However, social media could also be a source of disinformation and spread of inaccurate or incomplete information [10]. This is made worse by the ease to which users can disseminate information through various social media platforms without any third-party verification [7]. Misinformation causes mental health disorders such as panic attacks and anxiety disorders, and undermines efforts to curb the COVID-19 pandemic [7, 11, 12]. This Viewpoint suggests pragmatic methods through which social media can be harnessed to engage the public in the management of the ongoing COVID-19 pandemic in Cameroon.

### The role of social media in engaging the community while physical distancing

According to Cameroon´s situation report of 1 June 2020 on COVID-19, strategies taken to sensitise and engage the public involve the use of community health workers and volunteers to communicate the risk of transmission of the virus and methods of prevention in travel agencies, markets, and other social places [4]. Other strategies for community engagement involve inviting experts overseeing the COVID-19 pandemic in Cameroon to question and answer sessions on the evolution of COVID-19 in Cameroon, hosted by the local media. These sessions focus on transmission dynamics, diagnosis, management, and measures of prevention of COVID-19 in Cameroon. The use of community health workers and volunteers for sensitisation and community engagement in outbreaks such as cholera, poliomyelitis, and Ebola is fundamental and effective. However, this measure requires a substantial investment of human and financial resources. In a rapidly evolving and highly contagious pandemic like COVID-19, where time and preventing mass gathering are of the essence, this measure is limited because it becomes difficult to sustain a dialogue between community health workers and the public. Integrating the use of social media as a platform for a well-organised and coordinated community or public engagement would be time and cost-effective while maximising material and human resources. Social media could assist in community engagement in regions affected by civil conflicts, such as the Northwest and Southwest regions, or terrorist attacks like the Far North region, where community health workers are likely to be attacked in attempts to engage the community.

We recommend the use of limited social media accounts at central and regional levels to disseminate COVID-19-related information and to collect feedback from the public. These social media accounts, at least for the ministry of health and some regional delegations of health, do exist but their usage could be optimised. Having central and regional accounts will help track the public engagement. Bloggers or volunteers with the required experience could be employed and trained to ensure the daily running of these accounts. It is vital to have a steady source of reliable information which the public can always refer to for guidance. It is important to identify the best media outlet to disseminate information depending on the content needing to be shared. Based on our observation, WhatsApp, Twitter, and Facebook are currently the most active social media platforms in Cameroon. These platforms provide numerous opportunities to engage journalists, trusted social media influencers, and the wider public.

There are features on certain social media applications which can be utilised to optimise their efficiency. It is possible to tag other Twitter and Facebook users to maximise the target audience reached with each post. Hashtag is an important feature on Facebook and Twitter that could help followers retrieve previous posts on related topics. Currently, the hashtags *#covid19cmr* and *#covid19cameroon* are the most frequently used on Facebook and Twitter. In addition, Twitter and Facebook Live could be used to update the public on the government´s strategies to curtail the pandemic, and address queries from the public. The applications have options for followers to react to and share a post within their networks.

To optimise the coverage of a post, it is important to encourage sharing. We recommend working with credible local organisations or influential individual accounts to share COVID-19 content aimed at engaging the public. Religious leaders and institutions form an influential group in Cameroon and connect with their faithful through various social media platforms. About 90% of the Cameroonian population identify as either Christians or Muslims [13]. Most religious leaders and institutions have recently engaged in online activities with their followers. These leaders and institutions could be contacted to work with the government to engage the public. Effectively engaging the community will require a unified and coordinated action from medical and non-medical organisations.

The socioeconomic impact of COVID-19 on the lives of Cameroonians cannot be overemphasised. It is important for official government social media pages to engage in a dialogue with the public to receive feedback on how the pandemic is affecting their lives, and the difficulties they face in adhering to the measures put in place by the government to limit transmission of the virus. This will help the government identify gaps in its policy to tackle the pandemic and to strengthen her response accordingly. Also, social media ensures the public has a voice to demand accountability from the government in the management of COVID-19 and promote the dissemination of reliable information.

### Debunking misinformation and disinformation

Misinformation involves the spread of information that is not backed by facts. Disinformation is an intentional distortion of information to misinform. Since the first recorded case of the COVID-19 pandemic, there has been an escalation of misinformation globally that has been primarily incited by social media; a phenomenon which the World Health Organisation (WHO) refers to as an infodemic [14, 15]. Lack of trust and transparency in government authorities by the public appears to be the main driver of misinformation in Cameroon. Delays in updating Cameroon´s situation on various government websites lead the public to rely on unverified information from non-government websites and social media accounts within their networks. Such information might be inaccurate or purposefully distorted for a variety of reasons, including political agenda [16]. Regular, accountable, and transparent updates on the local evolution of the pandemic are necessary to build public trust. Currently, there are irregularities in reporting the local evolution of the pandemic. Reporting of daily statistics should be communicated from a dedicated government account and not personal accounts. This avoids conflicts in reporting and ensures consistency and accountability. Interviews organised with experts actively involved in managing the disease in Cameroon could be live-streamed on various government social media, including YouTube and Instagram. A mobile application created to verify information and demystify myths on the pandemic could also go a long way to avoid misinformation.

Efforts could be coordinated to identify, understand, and disrupt sources of misinformation. Specific hashtags of the COVID-19 pandemic in Cameroon could be browsed through to identify misinformation and address it through social campaigns. For example, a local non-government Health Education and Research Organisation (HERO) Cameroon launched a social media campaign to combat COVID-19-related misinformation [17]. Combating misinformation related to COVID-19 is challenging due to limited reliable scientific evidence on the disease. However, there is rapidly growing scientific evidence on COVID-19. A dedicated section for COVID-19-related publication on Research Gate, a social platform for researchers to share and discuss scientific publications and ideas, revealed that over 36000 publications currently exist on the topic cutting across basic sciences, social and economic impact, public health, and therapeutics [18]. The overwhelming influx of scientific information, which is easily accessed by the public, means the governments must clarify misinterpretation of the evidence or misinformation from publications. As evidence of treatment against COVID-19 from ongoing clinical trials become available, the public needed to understand when a treatment is effective and when it has potential of causing harm. A good example is the recent findings of the RECOVERY (Randomised Evaluation of COVID-19 Therapy) Trial on the effectiveness of dexamethasone for the treatment of COVID-19 [19]. Dexamethasone reduced the risk of 28-day mortality by one-third and one-fifth among patients admitted for COVID-19 and requiring invasive mechanical ventilation and those requiring supplemental oxygen without invasive mechanical ventilation, respectively [19]. There was no evidence of mortality benefits among those not needing any form of oxygen with a tendency towards harm (relative risk = 1.22 [95% confidence interval 0.93 to 1.61]; p = 0.14) [19]. With dexamethasone being a ubiquitous drug, the benefits and potential harm needs to be clearly communicated to the Cameroon public to prevent misinterpretation of the results and misuse use of the drug. These communications can easily be made through social media.

Information targeted towards the public needs to be succinct and reader-friendly. At the same time, information shared with the public has to be complete, practical, and if necessary, repetitive to prevent misinterpretation. Cameroon being a bilingual country means information should be communicated in English and French. Therefore, it is imperative that information is carefully translated from one language to another with the help of experts to prevent misinformation. The use of infographics or video communications are efficient ways to improve comprehension of the information being transmitted [20]. Moreover, the public should be directed on where to find stream-lined information on the management of COVID-19 in Cameroon, and the measures of prevention put in place by the Ministry of Health. The Ministry of Health dedicated a website for the fight against COVID-19, with an extensive section on frequently asked questions about the disease [21]. The link to this website can be disseminated through various social media platforms to direct the public toward reliable information. The frequently asked questions could be communicated to the public using infographics. In addition, the public could be directed to websites with reliable resources on COVID-19 for public education such as the WHO´s website.

The newness of COVID-19 has meant that it has become associated with social stigma, people fear the unknown and this fear can translate to fear of those around them. The stigma is worsened by the propagation of misinformation. Social stigmatisation of COVID-19 has significant implications in curbing the burden of the disease especially in developing countries like Cameroon where stringent social distancing measures like lockdowns are impractical. Stigmatisation can drive infected persons into denial because of fear of being discriminated against; meaning those infected with COVID-19 continue their daily activities, potentially contaminating others in the process. Stigmatisation can cause late presentation to care and discourage adherence to good prevention practices like wearing masks in public places. Destigmatising COVID-19 is crucial for the success of public health measures to contain the disease. Social media could be used to improve the public´s knowledge on COVID-19, break the myths about COVID-19, show empathy towards persons diagnosed with COVID-19 and their families, and support health care workers with higher risk of infection.

### Developing a culture of preparedness

As the health system of Cameroon becomes overwhelmed with the evolution of the pandemic, preventing infection of the already limited number of healthcare personnel should be an utmost priority. Infection of healthcare workers has significant repercussions on the COVID-19 response. On the one hand, infection of healthcare workers means a reduction in the capacity of the healthcare team leading to overburden and poor performance of the health system. On the other hand, healthcare workers are at risk of transmitting the virus to high-risk patients, leading to COVID-19 related hospital fatalities. Healthcare workers, if not protected would play an important role in the community transmission of SARS-CoV-2 [22]. Of the 6752 cases of COVID-19 reported on 1^st^ June 2022, 264 (3.9%) were healthcare workers [4]. There are restricted number of accredited COVID-19 management centres in Cameroon with personal protective equipment to prevent infection of frontline healthcare personnel. Efforts are being made to increase the number of management centres and across all ten regions of Cameroon. The influx of patients with symptoms of COVID-19 needs to be channelled away from health facilities ill-equipped for management COVID-19 towards government accredited COVID-19 management centres. To achieve this, information on when to consult a doctor, available phone contacts per region or district, when and where to get tested, what to do with the results, and where to receive care have to be clearly communicated to the public and updated as more management centres become available. Social media platforms can be used to help communicate this information to the public.

The website of the Ministry of Public Health of Cameroon dedicated for the management of COVID-19 contains a link to an online screening tool for patients who suspect they are experiencing symptoms of COVID-19 [21]. This tool captures information on the severity of the symptoms and location of the patient using a GPS tracker; and then uses this information to direct the patients to a suitable location to receive care. Social media could be used to increase public awareness on the availability of such a resource (Table 1).

**Table 1:** main axes of the use of social media to manage COVID-19 in Cameroon

Engaging the public while physical distancing	Use of central and regional social media accounts dedicated to fight against COVID-19
	Provide a steady source of reliable information to the public
	Identify the most used social media outlet to disseminate information to the public
	Leverage on the features of social media platforms to optimise the efficiency of public engagement
	Work with credible and influential individual accounts on social media to disseminate updates and key messages on COVID-19
Debunking misinformation and disinformation	Provide regular, accountable, and transparent updates on local evolution of the pandemic through a dedicated government account
	Clarify misinterpretation of evidence or misinformation from scientific publications
	Develop mobile application to verify information and demystify COVID-19-related myths
	Provide succinct and reader-friendly COVID-19-related information to the public
	Direct the public towards sources of streamlined information on COVID-19
Developing a culture of preparedness	Use social media platforms to orient the public towards accredited COVID-19 management centres

### Limitations of social media in managing the COVID-19 pandemic

Social media use is less likely to be an effective strategy to manage the COVID-19 pandemic in rural areas where there is limited access to electricity, internet connection, or smartphones. Social mobilisation using community health workers and volunteers is crucial to control the pandemic in rural areas and institutionalised groups such as prisoners. The ability for government agencies to engage the public through social media depends largely on how much the public can trust the government. Rebuilding the public´s trust in times like this demands strong political will to remain transparent, accountable, and committed.

## Conclusion

Social media is very popular in Cameroon, and despite it being used to disseminate misinformation regarding the COVID-19 pandemic, it can be a useful tool to fight the pandemic in Cameroon. While this looks promising, we do not underestimate the fact that there are inequalities in the access to internet services in Cameroon and a high cost associated with an even suboptimal internet supply.
